# Supplementary material for: Crawling and Gliding: A Computational Model for Shape-Driven Cell Migration
Source: PLoS Comput Biol. 2015 Oct 21;11(10):e1004280. doi: 10.1371/journal.pcbi.1004280 (PMC4619082; doi:10.1371/journal.pcbi.1004280)
Supplement: S1 Code — (ZIP) [file pcbi.1004280.s012.zip › release/tst/doc/html/hierarchy.html]

Tissue Simulation Toolkit: Class Hierarchy


Tissue Simulation Toolkit
 0.1.4.1


- Main Page
- Namespaces
- Classes
- Files

- Class List
- Class Hierarchy
- Class Members

Class Hierarchy

Go to the graphical class hierarchy

This inheritance list is sorted roughly, but not completely, alphabetically:

[detail level 12]

|  |  |
| --- | --- |
| Cell |  |
| CellularPotts |  |
| co |  |
| Dir |  |
| Dish | The virtual Petri dish |
| Graphics | API for Graphics windows |
| QtGraphics |  |
| X11Graphics | X-Windows implementation of Graphics interface |
| Info | Enables interactive querying of the simulation |
| li |  |
| Parameter |  |
| PDE |  |
| Point |  |
| **QWidget** |  |
| QtGraphics |  |


---

Generated on Thu Aug 14 2014 22:04:01 for Tissue Simulation Toolkit by  

 1.8.6
